# Similar COVID-19 incidence to the general population in people with opioid use disorder receiving integrated outpatient clinical care

**DOI:** 10.1016/j.dadr.2022.100027

**Published:** 2022-01-15

**Authors:** Gabriel Vallecillo, Francina Fonseca, Lina Oviedo, Xavier Durán, Ignacio Martinez, Alexandra García-Guix, Claudio Castillo, Marta Torrens, Santiago Llana, Albert Roquer, Maria de la Cabeza Martinez, Sandra Aguelo, Irene Canosa, COSG Group (COVID-19 and Opioids Study Group)

**Affiliations:** a Institut de Neuropsiquiatria i Addiccions, Hospital del Mar, Barcelona, Spain; b Addiction Research Group (GRAd), Neuroscience Research Program, Hospital del Mar Medical Research Institute (IMIM), Barcelona, Spain; c Universitat Pompeu Fabra, Barcelona, Spain; d Universitat Autònoma de Barcelona (UAB), Cerdanyola del Vallès, Spain

## Abstract

**Introduction:**

During the COVID-19 pandemic, limited access to health care augmented COVID-19 risk in subjects with opioid use disorder (OUD). The aim of the study was to compare COVID-19 incidence in individuals with OUD receiving continuous clinical care with that of the general population.

**Methods:**

A prospective cohort study was carried out from March 2020 to March 2021 comparing COVID-19 cumulative incidence of individuals presenting an OUD receiving integrated clinical care with that of an age-reference general population, in three public outpatient treatment centers for addiction in Barcelona, Spain.

**Results:**

Over the study period, 366 individuals received clinical care. Mean age: 48.2±8.9 years, 280 (76.5%) were men and 283 (77.3%) native Spanish. All subjects were on opioid agonist therapy. Prevalence of communicable diseases were: HIV infection in 109 (29.8%) and hepatitis C in 46 (12.6%). Psychiatric comorbidity was present in 207 (56.6%), and 119 (32.5%) had >1 chronic medical disease.

**COVID-19 was diagnosed in 10 patients:**

a cumulative incidence of 2,732 casesx100,000 people/year (C.I.95%: 1,318–4,967). There were no differences compared to the age-general population: 2,856 casesx100,000 people/year (C.I.95%: 2,830–2,880) (p=0.81). In the bivariate analysis, hypertension (5[50.0%] vs. 53[14.9%], p=0.01) and cardiovascular chronic diseases (2 [20.0%] vs. 8 [2.2%], p=0.03) were more prevalent in patients with OUD and COVID-19.

**Conclusions:**

Individuals with OUD who received integrated clinical care had a COVID-19 incidence comparable to the general population. Ensuring comprehensive healthcare is essential to prevent the clinical impact of COVID-19 on individuals with OUD.

## Highlights

• Opioid use disorder (OUD) patients in an integrated clinical approach had similar incidence of COVID-19 as general population.
• Cardiovascular diseases are more prevalent in (OUD) and COVID-19 infection.
• Comprehensive healthcare is essential in OUD to prevent clinical impact of COVID-19.

## 1 Introduction

The intersection between the opioid crisis and the coronavirus (COVID-19) pandemic is augmenting the negative effects of both in individuals with opioid use disorders (OUD) (Becker and Fiellin, 2020, Khatri and Perrone, 2020; Alexander et al., 2020). Those with OUD are more vulnerable to the social and economic stressors related to COVID-19, including social distancing, quarantine, fear of illness, and unemployment (Becker and Fiellin, 2020, Khatri and Perrone, 2020; Alexander et al., 2020). As a result, increased opioid use, risk of relapse for those in recovery, along with a rise in non-fatal/fatal overdoses have been observed (Khoury et al., 2021; Pines et al., 2021; Slavova et al., 2020; Rogers et al., 2020).

Moreover, they are at increased risk of COVID-19, and its more serious complications, due to a number of reasons (Wang et al., 2021a; Qeadan et al., 2021). Overcrowded living facilities such as shelters for the homeless, prisons, and densely-populated neighborhoods are high-risk environments for coronavirus transmission (Wang et al., 2020; Kinner et al., 2020). Recommendations to prevent the transmission and spread of COVID-19, including social isolation and quarantine, are difficult to maintain as drug-seeking and high-risk behaviors, such as sharing drug-using paraphernalia (Ksobiech, 2006) facilitate close personal contact. Moreover, the higher prevalence of comorbidities in aging individuals with OUD, compared to the general population, such as cardiovascular diseases, chronic respiratory diseases, diabetes, obesity, and cancer (Bahorik et al., 2017; Ejaz et al., 2020; Fareed et al., 2013; Salter et al., 2011), increases vulnerability to COVID-19 and is associated with more severe COVID-19 symptoms, complications, and fatalities (Fang et al., 2020; Gasmi et al., 2021; Izcovich et al., 2020). Besides which, substances of abuse have negative effects on various tissues and systems, including respiratory and immunologic ones (Stefanidou et al., 2011; Plein and Rittner, 2018; Radke et al., 2014), predisposing individuals to COVID-19 infection.

One of the main reasons, however, for such increased vulnerability to COVID-19 has been the reduced access to health care and recovery support services for individuals with OUD during the pandemic (Dunlop et al., 2020; Radfar et al., 2021; Stowe et al., 2020). Patient accessibility to treatment services and medications for OUD have been hindered by lockdown policies and the restricted schedules of treatment centers (Dunlop et al., 2020; Radfar et al., 2021; Stowe et al., 2020). Moreover, the adaptive capacities of the medical systems to the epidemic, reducing or closing outpatient services to redirect staff and other resources to manage acute COVID-19 cases, have resulted in suboptimal care for individuals with OUD (Dunlop et al., 2020; Radfar et al., 2021; Stowe et al., 2020). In this regard, public outpatient centers for drug addiction treatment and harm reduction centers were declared essential services in Spain during the pandemic. A measure that was carried out to ensure continuous clinical care, which could reduce COVID-19 incidence among individuals with OUD. The centers offer holistic, patient-centered clinical care through a multidisciplinary team composed of an addiction psychiatrist, addiction physician, and social worker.

Data, however, are lacking about the effectiveness of providing continuous clinical care for these subjects and the risk of COVID-19. The aim of the study was therefore to compare COVID-19 incidence in individuals with OUD receiving integrated care in three outpatient treatment centers for drug addiction, with that of a reference population during the pandemic in the Barcelona area (Spain).

## 2 Material and methods

This prospective cohort study was conducted in three outpatient treatment centers for drug addiction (CAS: Centro de Atención y Seguimiento a las Drogodependencias: Drug Addiction Attention and Follow-up Centre) located in or adjoining Barcelona, Spain: CAS Barceloneta, CAS Fòrum Sant Martí, and CAS Santa Coloma. The centers belong to the public health system and receive individuals from two different districts of Barcelona and another from the metropolitan area, respectively.

Subjects came voluntarily to the centers requesting substance use treatment. They were either self-referred or referred by their primary care physician or medical specialist. Access was free of charge, and individuals signed an informed consent to be included in the clinical programs. The main characteristics were: clinical management with individual counselling to encourage drug abstinence; health education, mainly harm-reduction information; methadone and buprenorphine-naloxone dosages provided as required (no restrictions on upper limit); and no restriction on treatment duration. Forced discharge occurred only as a result of patients' violent behaviour or drug trafficking.

The multidisciplinary care team included an addiction psychiatrist, a psychologist, an addiction physician, a social worker, and addiction nurses. According to individual characteristics, this team designed the most suitable drug abuse treatment modality for each case (detoxification, opioid agonist therapy, inpatient detoxification referral, and residential treatment). The subjects were monitored monthly at multidisciplinary sessions where the medical and psychosocial issues for each subject were discussed. The physician was a specialist in internal medicine and took care of the subjects’ health problems, in particular COVID-19, HIV-1 infection, tuberculosis prophylaxis or treatment, and hepatitis C co-infection. Antiretroviral therapy for HIV and hepatitis C was free of charge, dispensed monthly at the center, and self-administered or under direct supervision depending on the agreement arrived at between patient and physician. The nurses dispensed methadone, performed blood extractions, supervised urine substance screening tests, and were responsible for the needle-syringe exchange programme and harm reduction courses. The social services identified the patient's issues in order to find additional amenities and resources for them. In addition, they counseled patients through motivational interviewing to consider changing their problematic substance-using behavior, and supported them and their families. More details on the functioning of outpatient treatment centers for drug addiction (CAS) have been published elsewhere (Torrens et al., 2013).

From the beginning of pandemic in Spain, the centers remained open, and regular staff and all medical appointments were maintained except for group therapeutic activities which were cancelled. New admissions, mainly for OUD were encouraged by coordination with the nearest harm-reduction centers. Weekly drug urine tests were cancelled during the strict lockdown period, but performed again in May 2020 to assess the evolution of drug use.

Additional measures were incorporated into the clinical care routine of the centers including extending schedules and increasing home deliveries for opioid agonist therapy and other pharmacological treatments. Moreover, telemedicine and teletherapy services, COVID-19 prevention counseling, and provision of face-masks were implemented.

At each visit to the center, individuals were checked for symptoms and temperature. Assistance for symptomatic patients was coordinated with the reference hospital to carry out a real-time polymerase chain reaction for SARS-CoV-2 and complementary examinations. Depending on the COVID-19 severity, patients were admitted to hospital or, for milder cases, sent to external medical centers to complete quarantine.

At the end of the study period, patients were requested to voluntarily test for serological COVID-19 status through the determination of immunoglobulin IgG against N-protein of SARS-CoV2 by the qualitative chemiluminescent immunoassay LIAISON® SARS-CoV-2 TrimericS IgG, with 98.7% sensitivity and 99.5% specificity. Individuals who accepted be tested signed an informed consent.

For the purposes of the study, sociodemographic and clinical information were extracted from the patients’ medical records at the outpatient center and categorized to maintain anonymity where necessary. Opioid use disorders, and other psychiatric conditions were diagnosed according to the Diagnostic and Statistical Manual of Mental Disorders (5th Edition) [DSM-5]. Chronic diseases were diagnosed according to the International Statistical Classification of Diseases and Related Health Problems (ICD) [ICD-10] and grouped into seven conditions: hypertension, diabetes, respiratory, cardiovascular, renal, hepatic, and oncologic diseases.

The primary outcome of the study was the difference in the cumulative incidence rate of COVID-19 between individuals with OUD receiving integrated care and the age-reference population of Barcelona. Cumulative incidence was defined as new cases of COVID-19 over the study period, divided by the population at risk during the same period. Cumulative incidence from the reference population of Barcelona was extracted from the local COVID-19 Registry (Department of Health, Government of Catalunya, Spain; 2021). The secondary outcome was to assess the factors associated with COVID-19 among individuals with OUD and the prevalence of asymptomatic cases according to serological status, and measured as cases with positive IgG against N-protein of SARS-CoV2 divided by individuals with OUD who had had a serological test at the end of the study period.

Descriptive statistics were expressed as mean and standard deviation, or median and interquartile range, for the quantitative variables, and percentages for the qualitative ones. The Mann-Whitney test was used to compare quantitative variables, and the Fisher's exact test to compare categorical variable proportions. The confidence intervals for the cumulative incidences were calculated using the exact Clopper-Pearson method and the cumulative incidences were compared using the binomial test. Statistical analyses were performed with the free statistical R (3.5.2 version).

The study was approved by the local ethics committee (2020/9355/I, CEIC Parc de Salut Mar, Barcelona). All procedures performed in the study were in accordance with the ethical standards of the institutional research committee and with the 1964 Helsinki declaration and its later amendments.

## 3 Results

From March 18, 2020 (declaration of the Spanish state of alarm) to March 17, 2021, a total of 366 individuals received clinical care during a median of 19.3 (IQR;12-25) years at the three centers: 181 in CAS Barceloneta, 105 in CAS Santa Coloma, and 80 in CAS Forum. Social and substance use characteristics of the 366 subjects included in the study are shown in Table 1. Most of them were middle-aged, native Spanish men living at home. Two-thirds of the participants were single, half had criminal records, and a quarter of the samples was employed.

**Table 1:** Social and substance use characteristics of 366 people with opioid use disorders.

Characteristics	Total	Non-COVID-19	COVID-19	*p*
n	366	356	10	
Age^1^(years)	48.23 (8.89)	48.13 (8.84)	51.50 (10.5%)	0.30
Sex				
men	280 (76.5%)	273 (76.7%)	7 (70.0%)	0.71
Origin				
Spanish	283 (77.3%)	275 (77.2%)	8 (80.0%)	1
Education level				
primary school	240 (65.6%)	232 (65.2%)	8 (80.0%)	0.17
secondary school	113 (30.9%)	112 (31.5%)	1 (10.0%)	
post secondary school	13 (3.6%)	12 (3.4%)	1 (10.0%)	
Housing				
homeless	41 (11.2%)	41 (11.5%)	0 (0.0%)	0.67
shelter	27 (7.4%)	27 (7.6%)	0 (0.0%)	
home	298 (81.4%)	288 (80.9%)	10 (100%)	
Employment				
Employed	94 (25.7%)	89 (25.0%)	5 (50.0%)	0.14
unemployed	171 (46.7%)	169 (47.5%)	2 (20.0%)	
inactive	101 (27.6%)	98 (27.5%)	3 (30.0%)	
Marital status				
single	249 (68.0%)	244 (68.5%)	5 (50.0%)	0.11
couple	110 (30.1%)	106 (29.8%)	4 (40.0%)	
widow	7 (1.9%)	6 (1.7%)	1 (10.0%)	
Criminal recordsyes	187 (51.1%)	182 (51.1%)	5 (50.0%)	1
Substance use disorders				
heroine	366(100%)	356(100%)	10(10%)	1
cocaine	319 (87.2%)	309 (86.8%)	10 (100.0%)	0.37
cannabis	213 (58.4%)	205 (57.7%)	8 (80.0%)	0.20
alcohol	160 (43.7%)	153 (43.0%)	7 (70.0%)	0.11
Route administration				
injected	208 (56.8%)	201 (56.5%)	7 (70.0%)	0.40
nasal	82 (22.4%)	81 (22.8%)	1 (10.0%)	
pulmonary	61 (16.7%)	60 (16.9%)	1 (10.0%)	
oral	15 (4.1%)	14 (3.9%)	1 (10.0%)	
Agonist therapy				
methadone	302 (82.5%)	294 (82.6%)	8 (80.0%)	0.68
buprenorphine/naloxone	64 (17.5%)	62 (17.4%)	2 (20.0%)	
Drug urine test negative	232 (63.4%)	224 (62.9%)	8 (80.0%)	0.33

Data are presented as No. (%) unless otherwise indicated.

1 Data presented as mean ± standard deviation.

All the individuals with OUD included in the study had had heroin as the main opioid consumed before seeking treatment, and more than three-quarters were poly-substance users, mainly heroine plus cocaine. All the participants had been on opioid agonist therapy for a median of 19.3 (IQR: 12-25) years, and slightly more than half of them had negative results in drug urine tests.

The clinical characteristics of the individuals with OUD are shown in Table 2. The simultaneous diagnosis of substance use disorders (SUD) and other psychiatric conditions (dual diagnosis) was present in just over half of them: 92 (44.5%) had personality disorders, 48 (23.2%) affective disorders, 44 (21.3%) psychotic disorders, and 23 (11.1%) anxiety disorders.

**Table 2:** Clinical characteristics of 366 people with opioid use disorders.

Condition	Total	Non-COVID-19	COVID-19	*p*
Dual diagnosis	207 (56.6%)	202 (56.7%)	5 (50.0%)	0.75
HIV infection	237 (64.8%)	229 (64.3%)	8 (80.0%)	0.50
Hepatitis C infectionIgG antibodies	237 (64.8%)	229 (64.3%)	8 (80.0%)	0.50
RNA	46 (12.6%)	43 (12.1%)	3 (30.0%)	0.11
Hypertension	58 (15.8%)	53 (14.9%)	5 (50.0%)	0.01
Diabetes	27 (7.4%)	26 (7.3%)	1 (10.0%)	0.54
Liver chronic diseases	24 (6.6%)	24 (6.7%)	0 (0.0%)	1.00
Respiratory chronic diseases	57 (15.6%)	54 (15.2%)	3 (30.0%)	0.19
Cardiovascular chronic diseases	10 (2.7%)	8 (2.2%)	2 (20.0%)	0.02
Kidney chronic diseases	11 (3.0%)	10 (2.8%)	1 (10.0%)	0.26
Cancer	10 (2.7%)	10 (2.8%)	0 (0.0%)	1.00

Data are presented as No. (%) unless otherwise indicated.

Abbreviations: RNA, ribonucleic acid

All the subjects living with HIV were on antiretroviral therapy with a median CD4 cell count of 529 (IQR: 364-744) cells/mm^3^, and 93 (85.3%) had suppressed RNA-VIH-1. About two-thirds of them had serological IgG antibodies against hepatitis C, but only 46 (19.4%) had active hepatitis C infection.

Chronic medical disease was observed in 119 (32.5%) participants, mainly hypertension and respiratory conditions. The prevalence of any chronic medical disease was higher in subjects > 50 years (79 (51.6%) vs. 40 (18.7%), p<0.01)).

During the study period, 37 symptomatic subjects were tested for COVID-19, 10 of whom were positive. The cumulative incidence of COVID-19 in individuals with OUD was 2,732 cases x 100,000 people/year (C.I.95%: 1,318 – 4,967). There was no difference compared to the age-general population of reference: 2,856 cases x 100,000 persons/year (C.I.95%: 2,830 – 2,880) (p = 0.81).

In the bivariate analysis, hypertension (p = 0.01) and cardiovascular diseases (p = 0.02) were more prevalent in individuals with COVID-19.

One-hundred and fourteen subjects, who had not had COVID-19, agreed to be serologically tested. Only six patients had positive IgG against nucleocapsid, resulting in a 5.2% prevalence of asymptomatic COVID-19 (C.I.95%: 1.2-9.4) among individuals with OUD.

## 4 Discussion

Our findings show that individuals with OUD on agonist therapy, and receiving an integrated clinical approach in an outpatient center, had similar COVID-19 incidence compared to the age-reference general population. In addition, like the general population, chronic medical conditions, such as hypertension and cardiovascular disease (Fang et al., 2020; Gasmi et al., 2021; Izcovich et al., 2020). were more prevalent in individuals with OUD presenting COVID-19.

The results of the present work contrast with the data obtained from an analysis of the electronic health records of more than 73 million patients at 360 United States hospitals, 7.5 million of whom had an SUD (Wang et al., 2021b). Patients with OUD, particularly African Americans and those who had been diagnosed with OUD within the previous year, had an increased risk of COVID-19 and its adverse outcomes. Whilst insurance types did not affect the risk of COVID-19, there were no data about engagement in substance use treatment in this study. Moreover, substance use diagnoses in the electronic health records were coded using SNOMED-CT terminology which differs from DSM-5 categories (Wang et al., 2021b).

The low impact of COVID-19 in our study could have been justified by the patients’ attitudes. SARS-CoV-2 infection can manifest itself as an asymptomatic or mild disease (Oran and Topol, 2021; Wiersinga et al., 2020), which patients might have confused with withdrawal syndromes. In fact, one in three individuals were current drug users during the pandemic. Nevertheless, the results of the serological tests reflected a low prevalence of asymptomatic infections, similar to the general population of reference (Lai et al., 2020; Ministry of Health, 2021). In addition, patients could have decided against going to the saturated hospital emergency departments during the epidemic (Perlini et al., 2020). All thirty-seven symptomatic patients of the study did, however, undergo a real-time polymerase chain reaction for SARS-CoV-2 and complementary explorations to exclude COVID-19 diagnoses.

An interesting aspect of COVID-19 is the role comorbidities play in the risk of infection. Patients with severe psychiatric illness, particularly adults with a schizophrenia spectrum disorder diagnosis, have been described as a population at high-risk of infection including its more severe forms (Lee et al., 2020; Li et al., 2020; Nemani et al., 2021; Wang et al., 2021b). Our results, however, showed no association of psychiatric comorbidities with COVID-19 incidence. Such a contradictory result may reflect the importance of an optimal clinical approach to mental health problems in order to avoid the risk of infection.

In contrast, and similar to the general population (Fang et al., 2020; Gasmi et al., 2021; Izcovich et al., 2020), non-psychiatric comorbidities, especially hypertension and cardiovascular disease, were more prevalent in patients with OUD and COVID-19. Such a finding is relevant as the population with OUD is aging (Armstrong, 2007; Khatri and Perrone, 2020; Han et al., 2020); and has a high prevalence of chronic medical diseases (Maruyama et al., 2013) highlighting the need to prioritize COVID-19 vaccination for individuals with OUD and comorbidities (Iversen et al., 2021).

The present study had some limitations. The sample was representative of a Spanish cohort of individuals with OUD (Gutierrez-Caceres, et al., 2019), with a high prevalence of psychiatric illness, homelessness, unemployment, criminal records, polysubstance use, and current substance use despite agonist therapy treatment. Nevertheless, an intrinsic selection bias could have existed in the inclusion of individuals who voluntarily came to the drug abuse center and used the services. Such subjects, who were the most concerned for their own medical health and more motivated to start a drug abuse program, may have been overrepresented among this cohort (Stowe et al., 2020). In addition, the number of COVID-19 infections was quite low, which limited the ability to detect predictors of infection. By contrast, a considerable number of individuals with OUD had follow-ups during a long study period to observe differences.

With respect to the HIV pandemic in Spain the provision of harm-reduction programs led to a progressive decrease in infection rates among individuals with SUD (Stowe et al., 2020). In a similar manner, our findings highlight the importance of ensuring and maintaining comprehensive healthcare to reduce the impact of the epidemic on individuals with OUD and indirectly serve as a preventative measure to reduce the spread of COVID-19 in the general population. Person-centered attention is mandatory due to the multiple clinical and social problems faced by these patients. Therefore, comprehensive health care for individuals with SUD should be included along with a multidisciplinary, team-based, and coordinated-care approach. This should include health care, SUD treatment, harm reduction services, and access to housing and financial support. Integrated models can achieve this by solving the substantial structural and interpersonal barriers when accessing care services. Evidence is, however, lacking for the optimal method of service integration which depends on settings, local health laws, and provider training in both HIV disease and SUD treatment.

In conclusion, individuals with OUD receiving an integrated clinical approach in an outpatient center for drug addiction had similar COVID-19 incidence compared to the reference general population. Ensuring optimal healthcare is essential to prevent the clinical impact of COVID-19 on this vulnerable population, particularly for those with chronic medical conditions.

## Author contributions

GV conceived and managed the COSG Group (COVID-19 and Opioids Study Group). GV designed the study. FF, LO, AG, IM and CC built up and updated the database. XD analyzed the database. GV wrote the manuscript and all authors provided critical feedback and contributed to the writing of the manuscript.

## Declaration of Competing Interest

The authors declare no conflict of interest and the funders had no role in the design of the study, data collection, analyses, and interpretation, in the writing of the manuscript, or in the decision to publish the results.
